# A Functional Link between the Histone Demethylase PHF8 and the Transcription Factor ZNF711 in X-Linked Mental Retardation

**DOI:** 10.1016/j.molcel.2010.03.002

**Published:** 2010-04-23

**Authors:** Daniela Kleine-Kohlbrecher, Jesper Christensen, Julien Vandamme, Iratxe Abarrategui, Mads Bak, Niels Tommerup, Xiaobing Shi, Or Gozani, Juri Rappsilber, Anna Elisabetta Salcini, Kristian Helin

**Affiliations:** 1 Biotech Research and Innovation Centre (BRIC), Centre for Epigenetics, University of Copenhagen, Ole Maaløes Vej 5, 2200 Copenhagen, Denmark; 2 Wilhelm Johannsen Centre for Functional Genome Research, Department of Cellular and Molecular Medicine, University of Copenhagen, Blegdamsvej 3, 2200 Copenhagen N, Denmark; 3 Department of Biochemistry and Molecular Biology, The University of Texas M.D. Anderson Cancer Center, Houston, TX 77030, USA; 4 Department of Biological Sciences, Stanford University, Stanford, CA 94305, USA; 5 Wellcome Trust Centre for Cell Biology, University of Edinburgh, Edinburgh EH9 3JR, UK

## Abstract

X-linked mental retardation (XLMR) is an inherited disorder that mostly affects males and is caused by mutations in genes located on the X chromosome. Here, we show that the XLMR protein PHF8 and a *C. elegans* homolog *F29B9.2* catalyze demethylation of di- and monomethylated lysine 9 of histone H3 (H3K9me2/me1). The PHD domain of PHF8 binds to H3K4me3 and colocalizes with H3K4me3 at transcription initiation sites. Furthermore, PHF8 interacts with another XMLR protein, ZNF711, which binds to a subset of PHF8 target genes, including the XLMR gene *JARID1C*. Of interest, the *C. elegans* PHF8 homolog is highly expressed in neurons, and mutant animals show impaired locomotion. Taken together, our results functionally link the XLMR gene *PHF8* to two other XLMR genes, *ZNF711* and *JARID1C*, indicating that MR genes may be functionally linked in pathways, causing the complex phenotypes observed in patients developing MR.

## Graphical abstract

**Graphical Abstract:**

**Figure:**
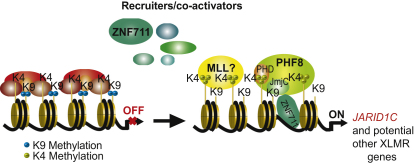

**Highlights:**

► PHF8 H3K9me2/me1 demethylase activity is lost in XLMR-associated PHF8 mutants ► PHF8 binds to most promoters with H3K4Me3, likely through its PHD finger ► The XLMR protein ZNF711 binds PHF8 and recruits PHF8 to many of its targets ► F29B9.2, a *C. elegans* PHF8 homolog, demethylates both H3K9me2 and H3K27me2

## Introduction

Individuals affected by mental retardation are characterized by impairment of intellectual functioning and deficits in adaptive behavior (American Psychiatric Association, 1994; Vaillend et al., 2008). X-linked mental retardation (XLMR) is an inherited disorder mostly affecting males and is often characterized by a complex phenotype. The XLMR conditions are grouped in two categories: a nonsyndromic form, in which mental retardation (MR) is the only clinical manifestation, and the syndromic form, in which MR is associated with a wide range of anomalies in various combinations, including biochemical disorders, neurological symptoms, skeletal abnormalities, and facial dysmorphies (Chiurazzi et al., 2008; Inlow and Restifo, 2004). XLMR is a genetic abnormality of the X chromosome caused by deletions, duplications, or inversions of large gene regions. However, monogenic mutations of the X chromosome are also a common cause of XLMR. XLMR genes are distributed over the entire X chromosome, and until now, around 82 XLMR genes have been identified and cloned. The XLMR gene products can be found in all cellular compartments and are involved in basic biological functions such as metabolism, DNA/RNA processing, protein synthesis, and cell-cycle regulation. However, a major part of the genes is involved in signal transduction and transcriptional regulation.

**Figure 1:**
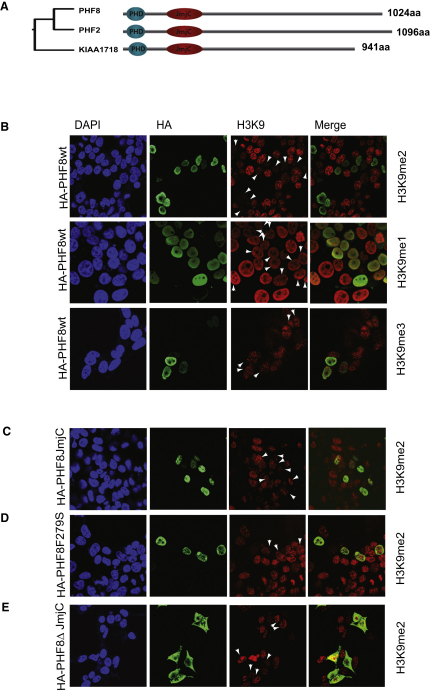
PHF8 Demethylates H3K9me2 In Vivo (A) Phylogenetic analysis and schematic representation of the human PHF family. Alignment of the proteins and construction of the phylogenetic tree were conducted using the ClustalW (http://align.genome.jp). Shown are jumonji C domain (JmjC) and plant homeotic domain (PHD). (B–E) (B) 293 cells were transfected with HA-tagged PHF8, (C) a PHF JmjC mutant, (D) a naturally occurring XLMR mutant with mutation in the JmjC domain (F279S), and (E) a C-terminal deletion (K177X) found in XLMR patients. The transfected cells were fixed, costained for the indicated histone modification and for the expression of the proteins (anti-HA), and analyzed by confocal microscopy. White arrows indicate cells expressing the tested protein. The cells were counterstained with DAPI to visualize cell nuclei.

The transcriptional regulatory factors consist of both putative DNA sequence-specific binding factors—among these, a number of zinc finger proteins, including ZNF41, ZNF81, ZNF674, and ZNF711 (Chiurazzi et al., 2008; Kleefstra et al., 2004; Lugtenberg et al., 2006; Shoichet et al., 2003; Tarpey et al., 2009)—and a variety of transcriptional cofactors modulating chromatin structure. For the latter, two of the XLMR proteins, JARID1C and PHF8 (Iwase et al., 2007; Jensen et al., 2005; Laumonnier et al., 2005; Tahiliani et al., 2007), belong to the JmjC group of proteins. This protein family has 27 members, and several of these have recently been shown to be histone lysine demethylases playing important roles in development and differentiation (Cloos et al., 2008; Klose and Zhang, 2007; Lan et al., 2008). Based on alignments of the JmjC domain, the family can be divided into 11 subgroups. Seven of these groups have been shown to catalyze the demethylation of methylated forms of lysines and arginines on histones H3 and H4 (H3K4, H3K9, H3K27, H3K36, and H3 R2/H4 R3). Mutations of *JARID1C* cause syndromic XLMR (Jensen et al., 2005), manifested by microcephaly and mild dysmorphic features. JARID1C belongs to the JARID1 subgroup, which has intrinsic H3K4me3/me2 demethylase activity (Cloos et al., 2008). JARID1C interacts with the Co-REST complex and is involved in the regulation of certain neuronal genes (Tahiliani et al., 2007). The *PHF8* gene is located at Xp11.22. The enzymatic and gene regulatory function of PHF8 is at the present unknown. Both deletions and point mutations in the JmjC domain of PHF8 have been reported and are associated with a syndromic phenotype characterized by mild XLMR and cleft lip/palate (Abidi et al., 2007; Koivisto et al., 2007; Laumonnier et al., 2005). Based on the overall domain structure and sequence homology, PHF8 is a potential demethylase related to KIAA1718 and PHF2, a JmjC subfamily with no assigned enzymatic specificity (Figure 1A). Two closely related homologs are present in *C. elegans* showing conservation of the overall domain structure and of the primary amino acid sequence in the PHD and JmjC domains, indicating an evolutionary conserved role for these proteins (Figure S1A available online). Here, we show that PHF8 is an H3K9me2/me1 demethylase, which binds H3K4me3 through its PHD domain and localizes to the H3K4me3-positive region of transcriptionally active genes in vivo, including a number of XLMR genes. PHF8 interacts directly with another XLMR protein, ZNF711, and co-occupies the promoter region of common target genes, suggesting a role for this factor in the recruitment of PHF8. A *C. elegans* homolog of PHF8 exhibits intrinsic H3K9me2 and H3K27me2 demethylase activities and is strongly expressed in neurons and is required for normal locomotion. Mutation of this gene causes a global increase in H3K9me2 and H3K27me2 levels during development, specifically linking the enzymatic activity to histone methylation in vivo.

## Results

### PHF8 Is an H3K9me2/me1 Demethylase

To examine the catalytic activity of PHF8, we transfected an HA-tagged version of PHF8 into HEK293 cells and analyzed histone methylation by immunofluorescence. As shown in Figure 1B, ectopic expression of PHF8 led to a slight decrease in dimethylated H3K9 levels, whereas the H3K9me1 and H3K9me3 levels were not significantly affected. The quantification of the H3K9me2 results showed that the observed decrease is significant and that ectopic expression of an XLMR-associated mutant of PHF8 led to a slight increase in H3K9me2 levels (Figure S1B). Also, no significant changes were observed for global H3K4me2, H3K4me3, and H3K27me2 methylation (Figure S1C). To test for the specificity of the reduction in H3K9 levels, we generated a PHF8 mutant (H247G/I248S/D249G), in which two of the three amino acids in the JmjC domain that is believed to be involved in Fe(II) binding were mutated. When this mutant was ectopically expressed in HEK293 cells, no reduction in H3K9 methylation levels was observed. Moreover, we tested the activity of two XLMR-associated PHF8 mutant proteins: F279S, carrying a mutation in the JmjC domain (Koivisto et al., 2007) and a C-terminal truncated mutant (K177X) deleting the nuclear localization signal (Abidi et al., 2007). Both of these mutants were found to have lost the ability to demethylate H3K9me2, probably by the inactivation of the intrinsic enzymatic activity and, for the latter, delocalization of the protein to the cytoplasm (Figures 1C–1E). Taken together, these data suggest that the PHF8 is an H3K9me2 demethylase and that the loss of this activity is associated with the development of XLMR.

**Figure 2:**
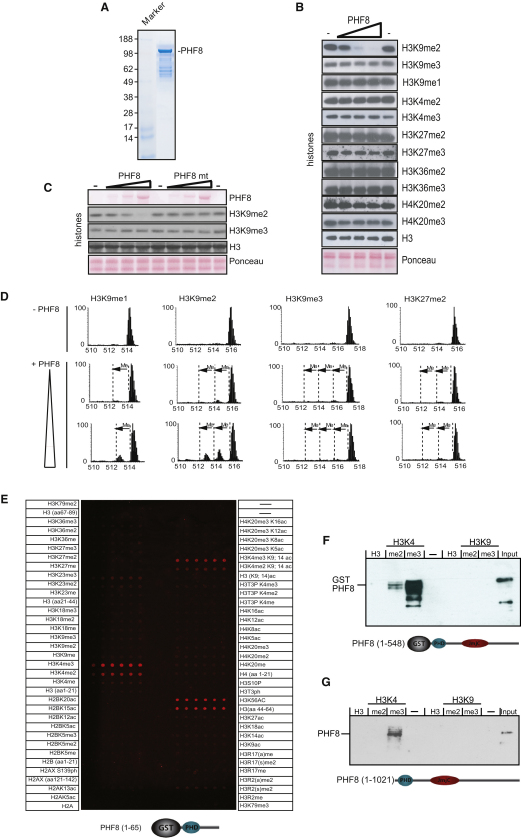
PHF8 Demethylates H3K9me2/me1 In Vitro and Binds to H3K4me3 (A) SDS-PAGE analysis of purified His-tagged recombinant PHF8 expressed in insect cells. Marker is a molecular weight standard. Samples were subjected to SDS-PAGE and stained using Coomassie blue. The arrow indicates the position of recombinant PHF8 (120 kDa). (B) Demethylation assays of histones incubated with 1.5, 4.5, and 12.5 μg of recombinant PHF8; bottom panels show Ponceau stain of the histones present in the assays. The middle panels show the reactions probed with the indicated antibodies and assayed by immunoblotting. (C) Demethylation assays of histones incubated with 1.5, 4.5, and 12.5 μg of recombinant PHF8 or a PHF8 JmjC mutant (H247R); bottom panels show Ponceau stain of the histones present in the assays. The middle panels show the reactions probed with the indicated antibodies and assayed by immunoblotting. (D) H3K9me3/me2/me1 and H3K27me2 peptides (2.5 μg) were incubated with or without recombinant PHF8 (0.5 or 1.5 μg) and analyzed by mass spectrometry. A shift in mass equivalent to one methyl group is indicated as “Me.” (E) Peptide array spotted with six replicates of modified peptides covering the large parts of histone H2A, H2B, H3, H2AX, and H4 modified by different degrees and combinations of methylation, acetylation, and phosphorylation. The modifications are listed in the panel. The array was incubated with a GST fusion protein harboring the PHD finger of PHF8, and the detection of bound protein was performed as described in the Supplemental Experimental Procedures. (F) In vitro binding experiment. A purified GST-PHF8 fusion protein (amino acids 1–548) containing the PHD and JmjC domain was incubated with biotinylated histone H3 peptides methylated at either the K4 or K9 position. After precipitation with Streptavidin-agarose, bound PHF8 was detected by SDS-PAGE and immunoblotting. (G) In vitro binding experiments. A lysate from HeLa cells was incubated with biotinylated histone H3 peptides methylated at either the K4 or K9 position. After precipitation with Streptavidin-agarose, bound PHF8 was detected by SDS-PAGE and immunoblotting.

To determine the intrinsic enzymatic specificity of PHF8, we affinity purified full-length recombinant his-tagged PHF8 from insect cells and tested its activity in vitro (Figure 2A). Incubation of purified calf thymus histones with increasing amounts of recombinant purified PHF8 (Figure 2B) led to a progressive loss of H3K9me2 methylation, whereas no detectable decrease in H3K9me1 and H3K9me3 levels was observed. In these assays, we did not observe any reduction in H3K27 methylation, despite the conservation of the four amino acids (ARKS) surrounding the methylated lysine of H3K9 and H3K27. Moreover, no changes of methylation were detected for methylated H3K4, H3K36, and H4K20, suggesting that the preferred substrate for PHF8 is H3K9me2. No enzymatic activity for PHF8 was detected when a histidine involved in iron binding located in the JmjC domain was changed to arginine (H247R) (Figures 2C and S2A), confirming the intrinsic enzymatic activity of PHF8. Moreover, we were not able to detect demethylation of nucleosomes as it has been observed before for other published histone demethylases (Iwase et al., 2007; Klose et al., 2006; Whetstine et al., 2006), suggesting that this is a poor substrate under standard assay conditions (Figure S2B). However, the accurate determination of histone demethylase specificity using histones as substrate can be limited due to the unknown quantitative differences between the different methylation states of the histones and antibody specificity.

Thus, to further characterize the enzymatic specificity of PHF8, we incubated increasing amounts of purified PHF8 with equimolar amounts of 40-mer peptides covering the histone H3 amino-terminal tail with the relevant methylation levels at K9(me3/me2/me1) and K27me2 position, respectively (Figure 2D). The reaction products were analyzed by mass spectrometry. In agreement with the specificity observed for histones, the H3K9me2 peptide was converted to the monomethylated and, to a lesser degree, the unmethylated form. Accordingly, the H3K9me1 peptide was also converted to the unmethylated peptide, albeit with less efficiency when compared to the H3K9me2 peptide. We did not observe any detectable demethylation of the H3K9me3 and the H3K27me2 peptides. However, at high concentrations of PHF8 catalyzing complete demethylation of the H3K9me2 peptide to its unmethylated form, we detected a low percentage conversion of the H3K27me2 peptide to the monomethylated form (Figure S2C). Collectively, these results indicate that the enzymatic specificity and order of substrate preference for PHF8 is H3K9me2 > H3K9me1 and that PHF8 only at very high concentrations has the ability to demethylate H3K27me2 in vitro.

### The PHF8 PHD Finger Binds to H3K4me3/me2

A common feature of the PHF family is the presence of amino-terminally localized PHD fingers. Recent studies have demonstrated that a subset of PHD fingers binds to histone tails and that the affinity is regulated by posttranslation modifications of the histone tails (Champagne and Kutateladze, 2009; Chan et al., 2009; Wysocka et al., 2006). These interactions are functionally important for regulating transcription. To examine whether the PHD finger of PHF8 binds to histone tails, we purified the PHF8 PHD domain as a GST fusion protein. The GST-PHD fusion was analyzed for binding to a peptide array covering large parts of H2A, H2B, H3, H2AX, and H4 modified by different degrees and combinations of methylation, acetylation, and phosphorylation (Figure 2E). The analysis demonstrated that the PHD domain binds directly to H3K4me3 and, to a lesser extent, H3K4me2. Of interest, the interaction was also observed when peptides, in addition to the H3K4me3, were modified by acetylation at the H3K9/K14 positions, modifications normally associated with transcriptionally active genes. Although we have not performed further experiments to validate the finding, we noted that the PHD domain of PHF8 was also able to bind to a peptide of H3 containing amino acids 44–64.

To examine the binding specificity further in the context of both the PHD and JmjC domain, a GST fusion protein encompassing the PHD and JmjC domain was incubated with biotinylated histone H3 peptides tri- or dimethylated at either the K4 or K9 positions (Figure 2F). Whereas no binding to peptides methylated at the K9 position was detected, a strong binding to H3K4me3 was observed, supporting the peptide array mapping data. Finally, endogenous PHF8 expressed in HeLa was shown to bind H3K4me3, whereas no binding was detected for the unmodified or H3K9me3/me2-containing peptides (Figures 2G and S2D). Together, these data demonstrate that the PHD finger of PHF8 can bind directly to H3K4me3-modified histone tails and that the JmjC domain, at least under the binding conditions, does not interfere with H3K4me3 or mediate stable binding to its substrate H3K9me2.

### PHF8 and H3K4me3 Colocalize at Target Genes

**Figure 3:**
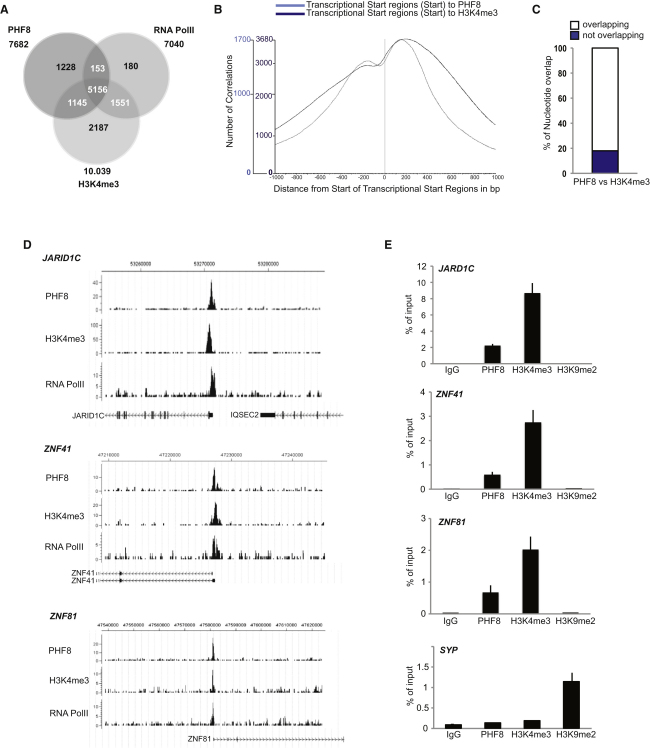
PHF8 Binds Target Genes in the H3K4me3-Positive Region Surrounding the TSS (A) Venn diagram showing the overlap and number of genes positive for PHF8, H3K4me3, and RNA pol II in human neuroblastoma SH-SY5Y cells. (B) Distribution of the distance between PHF8 (light blue) and H3K4me2 (dark blue) peaks and the transcription start sites (TSS). (C) PHF8 nucleotides that are also bound by H3K4me3. (D) Binding profiles of the XLMR genes *JARID1C*, *ZNF41*, and *ZNF81* for PHF8, H3K4me3, and RNA pol II obtained from ChIP-seq analysis; the chromosomal locations are indicated in the top of the panel according to hg18. The Y axes indicate the number of sequenced reads. (E) qChIP analysis of the XMLR genes *JARID1C*, *ZNF41*, and *ZNF81* in SH-SY5Y cells using antibodies against PHF8, H3K4me3, H3K9me2, and IgG control. The enrichment of the ChIP assays is shown as percentage of input. qChIP analysis of the gene coding for synaptophysin (*SYP*) using antibodies against PHF8, H3K4me3, H3K9me2, and IgG (control) was used as positive control for H3K9me2. Error bars represent SD; n = 3.

Genome-wide analysis of H3K4me3 has revealed that this chromatin modification is preferentially associated with transcription start sites (TSS) (Barski et al., 2007). The affinity of PHF8 for H3K4me3 could suggest that PHF8 will bind target genes around the TSS positive for H3K4me3. To determine the genome-wide binding sites of PHF8, we performed genome-wide location analysis of PHF8 in human neuroblastoma SH-SY5Y cells by high-resolution ChIP-seq (chromatin immunoprecipitation followed by DNA sequencing) analysis using antibodies specific for PHF8 and correlated this with the binding pattern of RNA polymerase II (RNA pol II) and H3K4me3. The total number of peaks detected for PHF8 based on 9.8 million sequence reads and 9.1 million reads for H3K4me3 after IgG normalization were 17075 and 19534, respectively. Annotation of the peaks to genes using the hg18 genome database revealed that 10039 genes were positive for H3K4me3 and 7682 genes were bound by PHF8 within ± 1000 bp of the TSS. The Venn diagram in Figure 3A illustrates the number of genes positive for PHF8 and H3K4me4 and demonstrates a major overlap of PHF8- and H3K4me3-positive genes (∼82%), whereas 35% of H3K4me3-positive genes are PHF8 negative. In general, PHF8 either colocalizes or partly overlaps with the regions positive for H3K4me3 and covers the putative TSS of the target genes. Analysis of the distance correlation between the peaks detected for PHF8 and H3K4me3 and transcriptional start sites demonstrates that both peaks are predominantly overlapping with the TSSs containing a common peak maximum at around +200 (Figure 3B). Furthermore, 82% of the nucleotides bound by H3K4me3 are also bound by PHF8 (Figure 3C). However, there is no strict correlation between PHF8 binding and H3K4me3 levels (compare, for instance, CXXC1 and MBD1; Figure S3A). Of interest, ∼35% of the H3K4me3-positive genes are negative for PHF8, indicating that other factors in addition to the PHD domain of PHF8 might determine gene targeting. A major part of PHF8-bound genes (∼69%) is also RNA pol II positive, suggesting that they are either “poised” for transcription or are transcriptionally active. Because we observed a slight decrease in the binding of the PHD domain of PHF8, an H3K4me3 peptide containing K9Ac and K14Ac compared to H3K4me3 alone (Figure 2E), we speculated that histone hyperacetylation could influence the recruitment of PHF8 to target gene promoters. However, ChIP experiments using antibodies specific for H3K9ac, H3K14ac, and H3K4me3 showed that PHF8 is associated with genes that are positive for all three histone marks (Figure S3B). This result suggests that hyperacetylation of histones is not influencing the binding of PHF8 to target genes and further supports the notion that PHF8 target genes are transcriptionally active.

Because mutation of *PHF8* causes mental retardation, PHF8 might potentially be involved in the regulation of other XLMR genes. In SH-SY5Y cells, PHF8 bound a number of reported XLMR genes. Inspection of selected XMLR genes *JARID1C*, *ZNF41*, and *ZNF81* supported the localization of PHF8 at the H3K4me3-positive region surrounding the TSS site (Figure 3D). These targets were validated by qChIP (ChIP followed by real-time quantitative PCR), showing the binding of PHF8 and H3K4me3 (Figure 3E). These genes were also positive for RNA pol II, indicating active transcription (Figure 3D). Whereas the chromatin status in terms of methylation is unknown for these genes when inactive, all genes were negative for H3K9me2, suggesting a potential role for PHF8 in the elimination of this repressive chromatin mark. The colocalization of PHF8 and H3K4me3 indicates a role for PHF8 as a positive regulator of transcription by locally reducing H3K9me2 at the TSS. Unfortunately, the current available antibodies specific for H3K9me2 are not very potent in ChIP assays, and therefore, they do not work reliably in genome-wide location studies (unpublished observations; Barski et al., 2007). To test that the H3K9me2 antibodies can work in ChIP, we showed that H3K9me2 is enriched at the synaptophysin gene (*SYP*), which has earlier been reported to be H3K9me2 positive in SH-SY5Y cells (Ekici et al., 2008), and, importantly, it is not bound by PHF8. This result indicates that the lack of H3K9me2 at PHF8 target genes is due to the absence of the mark.

### PHF8 Binds to the XLMR Gene Product ZNF711

**Figure 4:**
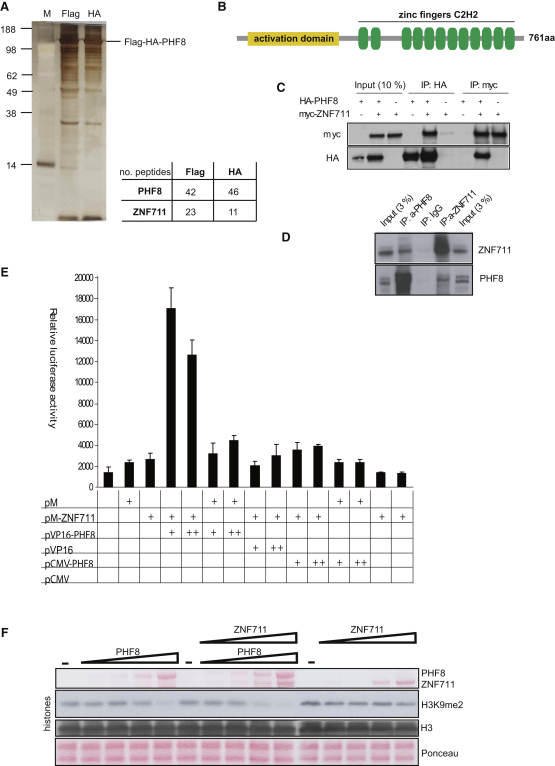
PHF8 Interacts with ZNF711 (A) Flag-HA tandem purification of PHF8 complexes. Silver-stained SDS-PAGE analysis of Flag- and Flag-HA-purified complex from 293 cells. M, molecular weight marker; F, Flag-eluate; H, Flag-eluate further purified by HA-affinity chromatography. The eluted material was analyzed in bulk by mass spectrometry. The numbers of peptides identified for PHF8 and ZNF11 for single-step Flag purification or double Flag-HA purification are shown. (B) Schematic representation of ZNF711. Shown are the putative activation domain and ZNF, Cys-His zinc finger domain. (C) Coimmunoprecipitation of PHF8 and ZNF711. Phoenix cells were transfected or cotransfected with the expression vectors pCMV-HA-PHF8 and pCMV-Myc-ZNF711 and were immunoprecipitated using antibodies against the HA-tag or Myc-tag as indicated. The precipitates were analyzed by SDS-PAGE followed by western blotting using antibodies against the Myc-tag or HA-tag. (D) Coimmunoprecipitation of endogenous PHF8 and ZNF711. HEK293 cells were lysed and immunoprecipitated with the indicated antibodies against ZNF711 and PHF8. The precipitates were analyzed by SDS-PAGE followed by western blotting using antibodies against ZNF711 and PHF8. (E) Interaction of PHF8 and ZNF711 in a mammalian two-hybrid assay. U2OS cells were transfected with a pGAL-Luc reporter plasmid containing the luciferase gene driven by the adenovirus E1B minimal promoter (TATA) fused to five upstream GAL4-binding sites or cotransfected with the expression constructs indicated in the bottom of the panel. For correction of transfection efficiency, pCMV-lacZ was included in all assays, and luciferase activity was normalized to β-galactosidase activity. All experiments were performed in triplicate and reproduced at least three times. Error bars represent SD; n = 3. (F) Demethylation assays of histones incubated with 1.5, 4.5, and 12.5 μg of recombinant PHF8 alone and/or in the presence of 1, 3, and 9 ug of recombinant ZNF711; bottom panels show Ponceau stain of the histones present in the assays. The middle panels show the reactions probed with the indicated antibodies and assayed by immunoblotting.

The lack of PHF8 binding to all H3K4me3-positive genes and differential binding to H3K4me3-positive binding indicates that other factors influence PHF8 recruitment. To identify such recruiters/cofactors, we purified PHF8-interacting proteins by tandem affinity chromatography. Flag-HA-tagged PHF8 was stably expressed in human TREX 293 cells, and interacting proteins were purified from nuclear extracts by Flag- followed by HA-affinity chromatography (Figure 4A). Subsequently, interacting proteins were identified by mass spectrometry. A single protein, ZNF711, was specifically highly enriched in the affinity purification of PHF8-associated proteins. The *ZNF711* gene is located at Xq21.1-q21.2 and encodes a zinc finger protein with unknown function. It harbors an amino-terminal potential domain followed by 12 consecutive Zn-C2H2 domains potentially involved in transcriptional activation and sequence-specific DNA binding, respectively (Figure 4B). The gene is predominantly expressed in parts of the brain and retina in mice (Figure S4B). In humans, the gene is expressed in neural tissues and highly expressed in 293 cells and in neuroblastoma SH-SY5Y cells (Figure S4A). These cell lines also express high levels of PHF8 (Figures S4C and S4D). Recently, a mutation of the zinc finger region of ZNF711 was shown to be associated with mild mental retardation in patients resembling the MR phenotype observed for mutations of PHF8 (Tarpey et al., 2009). However, the cleft lip/palate phenotype associated with PHF8 mutations was not observed in these patients.

To verify the PHF8-ZNF711 interaction, we coexpressed HA- and Myc-tagged versions of PHF8 and ZNF711 and analyzed complex formation by coimmunoprecipitation. As shown in Figure 4C, immunoprecipitation of either HA-PHF8 or Myc-ZNF711 highly enriches the other factor, confirming the interaction. Consistent with this finding, endogenous ZNF711 was also immunoprecipitated from HEK293 cells by an anti-PHF8, and endogenous PHF8 was immunoprecipitated using an anti-ZNF711 antibody (Figure 4D). These results demonstrate that ZNF711 binds to PHF8 in vivo. Furthermore, a functional interaction between the two proteins was observed in a mammalian two-hybrid assay (Figure 4E). The strong interaction in these assays indicates that PHF8 and ZNF711 bind directly to each other and that ZNF711 could be a PHF8 recruiter/cofactor. To investigate whether ZNF711 influenced the enzymatic activity of PHF8 in vitro, we purified recombinant ZNF711 (Figure S4E) and titrated equimolar amounts of PHF8 into the histone demethylation assay. When compared to PHF8 alone, the inclusion of ZNF711 in the assay resulted in a slight increase in demethylation activity (Figure 4F). Whereas the interpretation of the assay is complex, partly due to the instability of purified PHF8 and the subtle effect of ZNF711, the results suggest that a ZNF711-PHF8 complex is active in vivo. In line with the lack of PHF8 activity on nucleosomes, PHF8 together with ZNF711 did not catalyze demethylation of nucleosomes in vitro (Figure S4F).

### ZNF711 Colocalizes with PHF8 on a Subset of PHF8 Target Genes

**Figure 5:**
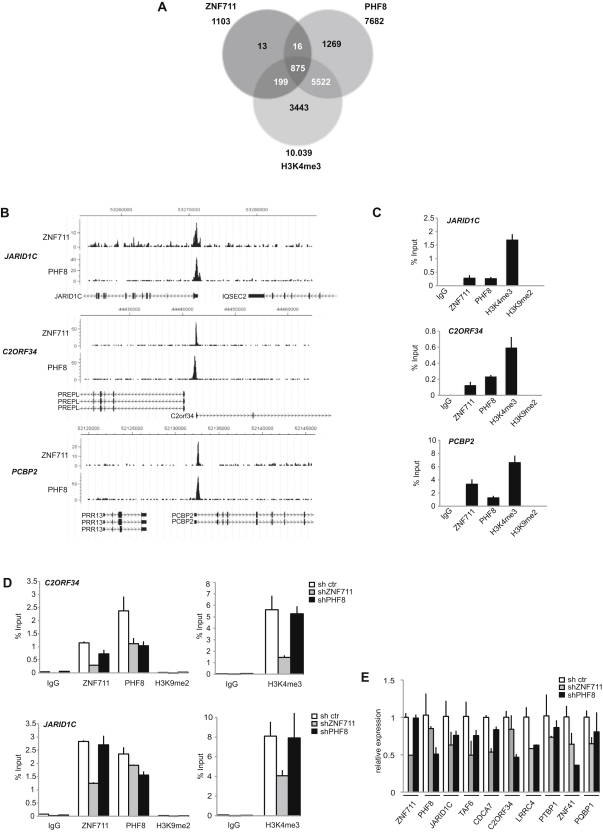
PHF8 Colocalizes with ZNF711 on Target Genes (A) Venn diagram showing the overlap and number of genes positive for ZNF711, PHF8, and H3K4me3 in human neuroblastoma SH-SY5Y cells. (B) Binding profiles of the ZNF711 target genes *JARID1C*, *C2ORF34*, and *PCBP2* for ZNF711 and PHF8 obtained from ChIP-seq analysis; the chromosomal locations are indicated in the top of the panel according to hg18. The Y axes indicate the number of sequenced reads. (C) qChIP analysis of ZNF711 target genes *JARID1C*, *C2ORF34*, and *PCBP2* in SH-SY5Y cells using antibodies against ZNF711, PHF8, H3K4me3, H3K9me2, and IgG control. The enrichment of the ChIP assays is shown as percentage bound of input. (D) qChIP analysis of ZNF711 target genes *JARID1C* and *C2ORF34* in HEK293 cells after shRNA-mediated ZNF711 knockdown using antibodies against ZNF711, PHF8, H3K4me3, and IgG control. (E) Expression analysis of ZNF711-PHF8 target genes after shRNA-mediated ZNF711 and PHF8 knockdown in HEK293 cells using real-time RT-qPCR. Error bars represent SD; n = 3.

To examine the role of the interaction between PHF8 and ZNF711 in gene regulation, we generated a specific antibody against ZNF711, and, based on 9.6 million sequence reads, a genome-wide map of ZNF711 DNA-binding sites in SH-SY5Y cells was obtained by ChIP-seq analysis. The 1875 binding peaks were detected, and annotation of the peaks to genes within 1 kb of the TSS using the hg18 genome database revealed that 1204 of the peaks mapped to 1103 genes positive for ZNF711. The overlap between ZNF711- and PHF8-bound genes were ∼80%, and the majority (∼97%) was H3K4me3 positive, as shown in the Venn diagram in Figure 5A. Similar to PHF8, ZNF711 bound its target genes in close vicinity of the TSS. Furthermore, genes strongly binding ZNF711 also bound high levels of PHF8, and the proteins showed overlapping or closely juxtaposed binding regions (Figure 5B). These results further support the finding that the two proteins interact. In contrast to PHF8, which binds to a number of XLMR genes in SH-SY5Y cells, ZNF711 only bound *JARID1C*. To validate the ChIP-seq mapping of ZNF711 and determine chromatin modifications, we analyzed the binding of ZNF711, PHF8, and the chromatin status for H3K4me3 and H3K9me2 for *JARID1C* and two highly scoring ZNF711 targets *C2orf34* and *PCBP2* by qChIP using primer sets covering the ZNF711 binding region (Figure 5C). The genes showed high enrichment for ZNF711, PHF8, and H3K4me3, whereas H3K9me2 methylation was not detected, indicating a role for PHF8 in the removal of this mark at TSS.

To understand whether ZNF711 is involved in the recruitment of PHF8 to target genes, we downregulated ZNF711 expression in 293 cells using shRNA and analyzed ZNF711 and PHF8 binding to *JARID1C* and *C2orf34*. As expected, the binding of both ZNF711 and PHF8 was reduced. In addition, the levels of H3K4me3 were reduced, further supporting a role for the complex in active transcription. An increase in H3K9me2 levels upon decreased binding of PHF8 to the analyzed gene loci was not observed. Moreover, analysis of H3K9me2 levels associated with *JARID1C* and *C2ORF34* 500 bp downstream and 1000 kb upstream of the transcription start site did not show a significant increase in H3K9me2 levels after knockdown of PHF8 (Figure S5A). As shown below, the PHF8 homolog in *C. elegans* F29B9.2 demethylates both H3K9me2 and H3K27me2, and we therefore tested whether knockdown of PHF8 could affect H3K27me2 levels at target genes even though PHF8 did not show activity toward this mark in in vitro and in vivo assays. H3K27me2 ChIP experiments did not lead to any detectable increase in H3K27me2 levels at the transcription start sites of *JARID1C* and *C2ORF34* upon knockdown of PHF8 (Figure S5B). To test whether redundancy with the other two related PHF8 homologs PHF2 and KIAA1718 might explain the lack of a change in H3K9me2 levels at target gene promoters, RNAi experiments against all three genes were performed. We did not observe any effect on H3K9me2 levels after single or combined downregulation of PHF8, PHF2, and KIAA1718 expression levels on the PHF8 target genes *JARID1C* and *C2ORF34* (Figures S5C and S5D).

To test whether the downregulation of ZNF711 and PHF8 would lead to a change in transcription of target genes, we tested the expression of nine different ZNF711/PHF8 target genes by qPCR following shRNA-mediated knockdown of ZNF711 and PHF8 expression. As shown in Figure 5E, the expression of all of the tested target genes was significantly decreased in 293 cells expressing shRNAs to ZNF711 or PHF8 (Figure 5E). A similar decrease in expression levels of ZNF711-PHF8 target genes was also observed in SH-SY5Y cells following siRNA-mediated inhibition of PHF8 expression (Figure S5F). In contrast, we did not observe any effect of inhibition PHF2 and KIAA1718 expression on the PHF8 target genes (Figure S5E).

### A PHF8 Homolog in *C. elegans* Has H3K9/27me2 Demethylase Activity and Modulates Coordinate Locomotion

**Figure 6:**
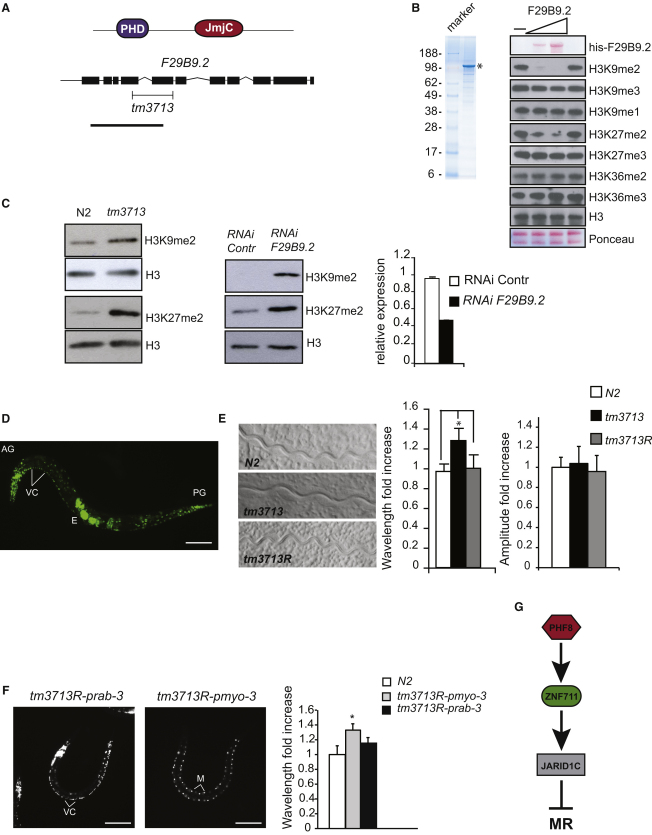
The *C. elegans* PHF8 Homolog F29B9.2 Is an H3K9me2 and H3K27me2 Demethylase Involved in Coordinated Movement (A) (Top) Schematic representation of the *C. elegans* F29B9.2 protein. PHD, plant homeodomain finger; JmjC, jumonji C domain. (Bottom) Genomic organization of the *F29B9.2* gene. Exons are indicated by black boxes. Black H-shaped line indicates the position of the *tm3713* deletion. Black line indicates the position of the fragment used for RNA interference. (B) SDS-PAGE analysis of purified His-tagged recombinant F29B9.2 expressed in insect cells. Marker is molecular weight standard. Samples were subjected to SDS-PAGE and stained using Coomassie blue. The arrow indicates the position of recombinant F29B9.2. Demethylation assays of histones incubated with 1.5 and 4.5 μg of recombinant F29B9.2; bottom panels show Ponceau stain of the histones present in the assays. The middle panels show the reactions probed with the indicated antibodies and assayed by immunoblotting. (C) Analysis of histone modifications in wild-type animals (N2), *tm3713* mutants, and *RNAi* (*F29B9.2*)-treated animals. (Left) Protein lysates from synchronized N2 and *tm3713* young adults were probed with the indicated antibodies. (Middle) Protein lysates from synchronized F1 *eri-1* adult worms treated with control or *F29B9.2(RNAi)* were probed with antibodies as indicated. (Right) The relative expression level of F29B9.2 mRNA in control and *RNAi-*treated animals, quantified by real-time RT-PCR. Error bars represent SD; n = 3. (D) Epifluorescent image of an adult hermaphrodite carrying the *F29B9.2::GFP* transgene. VC, ventral nerve cord; AG, anterior ganglia; PG, posterior ganglia; E, embryos. In (D) and (F), anterior is in the left; ventral, down. Scale bar represents 100 μm. (E) Abnormal movement on bacterial-seeded plates of *tm3713* mutant animals, rescued in *tm3713* carrying the *F29B9.2::GFP* transgene (*tm3713R*). The graphics show the measure of the wavelength and amplitude of N2, *tm3713*, and *tm3713R*. Values were normalized to the N2. Error bars in (E) and (F) represent standard deviation of the mean. Asterisks in the graphics indicate results different at p < 0.01 (Student's t test). The variations on amplitude are not significant (p > 0.05). (F) *tm3713* mutant animals expressing *F29B9.2* under neuronal (*rab-3*) and muscle (*myo-3*) promoters. Expression of *F29B9.2* in neurons restores wild-type locomotion. The graphic shows the measure of the wavelength of N2 and transgenic animals normalized to the values of the N2. (G) PHF8, ZNF711, and JARID1C are linked in a pathway required for suppressing mental retardation.

*C. elegans* has two closely related sequences, *F29B9.2* and *F43G6.6*, that have strong homology to the human PHF family (Figure S1). To identify the in vivo role of this class of demethylases, we characterized the function of *F29B9.2* (Figure 6A). Similarly to the human counterpart, recombinant full-length *F29B9.2* (cePHF8) demethylates H3K9me2 in vitro (Figure 6B). We also noticed that H3K27me2 was reduced in these conditions, suggesting that *F29B9.2* may have a dual specificity not identified in the human PHF8. To investigate the role of *F29B9.2* in vivo, we analyzed a mutant allele of *F29B9.2*, *tm3713*, generated by the Japanese National BioResource Project. The *tm3713* allele carries a deletion of 575 bp spanning the fourth and fifth exon of *F29B9.2* (Figure 6A), and the predicted protein, if produced, would therefore lack the PHD domain. In agreement with the results obtained in vitro, protein lysates from mutant animals showed an increase in global levels of H3K9me2 and H3K27me2, compared to wild-type animals (Figure 6C). Furthermore, inhibition of *F29B9.2* expression by RNA interference also led to an increase in H3K9me2 and H3K27me2 levels. These results strongly suggest that *F29B9.2* is an H3K9me2 and H3K27me2 demethylase.

To obtain information regarding the functional role of *F29B9.2* in *C. elegans*, we analyzed the expression pattern of *F29B9.2* by generating a translational fusion between the *F29B9.2* genetic locus and GFP. Transgenic animals bearing *F29B9.2::GFP* in an extrachromosomal array exhibited strong nuclear fluorescence in neurons (Figure 6D), even though a few other cells (some muscle, intestinal, and hypodermal cells) showed a faint fluorescence. The neuronal expression pattern together with the fact that human *PHF8* is a XLMR gene prompted us to test the deletion allele *tm3713* for phenotypes related to the function of the nerve system. Of interest, the *tm3713* allele exhibited defects in body movement. Wild-type N2 animals moved by alternating dorsal and ventral flexions along the body length, producing a regular sinusoidal track on bacteria (Figure 6E). In contrast to this, the track pattern left by *tm3713* was irregular, with increased wavelength (distance between successive peaks of a wave) but unchanged amplitude of the wave. This phenotype is not due to increased length of the animal body compared to wild-type (data not shown). Strikingly, reintroduction of *F29B9.2* gene in *tm3713* background (*tm3713R*) restored the wild-type phenotype, demonstrating that the locomotion defect is specific to the loss of *F29B9.2*. Moreover, re-expression of the gene in mutant background under a pan-neuronal promoter (*rab-3*) (Nonet et al., 1997), but not under a muscle promoter (*myo-3*) (Fire and Waterston, 1989), rescued the phenotype associated with loss of *F29B9.2* (Figure 6F). We did not observe other phenotypes related to neuronal function, such as pumping, dye filling in sensory neurons, egg laying, sensitivity to levamisole, aldicarb, and serotonin in the *tm3713* mutant allele (data not shown). However, in conclusion, our data suggest that *F29B9.2* is important for normal neuronal function in *C. elegans*.

## Discussion

More than 82 genes have been linked to XLMR, and this number is expected to increase in the coming years (Chiurazzi et al., 2008). XLMR is believed to affect 1 in 600 in the male population; however, despite the high prevalence, the complexity of the disease and the large number of genes associated with it have hampered a better understanding of the molecular mechanisms leading to mental retardation. In this report, we have linked three previously mapped XLMR genes (Figure 6F), two of them uncharacterized, which are believed to have a causative role in XLMR (Abidi et al., 2007; Koivisto et al., 2007; Laumonnier et al., 2005; Tarpey et al., 2009). We have demonstrated that PHF8 is an H3K9me2/me1 demethylase and that it is bound very close to transcriptional start sites of a large number of active or poised genes. Of interest, the large majority of PHF8-bound genes are also associated with H3K4me3, and the PHF8 and H3K4me3 binding profiles are strikingly similar. These results suggest that the H3K4me3 mark could be involved in the recruitment of PHF8 to active promoters, and accordingly, we show that the PHD domain of PHF8 binds directly to H3K4me3. However, the demonstration that PHF8 is not bound to all H3K4me3-positive genes suggests that this mark is not sufficient for PHF8 recruitment and that other factors are involved. In agreement with this, we found that PHF8 directly interacts with ZNF711, suggesting a role for this factor in the recruitment of PHF8 to common promoters. Thus, the interaction between ZNF711 and PHF8 may instead stabilize the association of PHF8 with the histone tail either before or after H3K4me3 methylation and catalyze the efficient removal of H3K9me2.

Dimethylation of H3K9 has mainly been associated with transcriptionally inactive genes (Peters et al., 2003) and may catalyze gene repression by recruitment of repressive complexes interacting with the H3K9 mark. Alternatively, H3K9me2 may hinder acetylation of the H3K9 position, a histone modification present at the TSS site of active genes. Consistent with a role of PHF8 as an H3K9me2 demethylase, PHF8 is bound to active genes lacking the H3K9me2 mark. Even though our results show that both PHF8 and ZNF711 contribute to the activation of target genes, including *JARID1C*, we have so far been unable to detect an increase in H3K9me2 levels upon PHF8 or ZNF711 downregulation. Whereas transcription factors other than ZNF711 and PHF8 are probably important for transcription at target promoters, this observation indicates that, in a static cellular system like the SH-SY5Y cells, recruitment of a specific H3K9me2 methyltransferase is not a default mechanism. Our data showing that inactivation of a *C. elegans* PHF8 homolog leads to a strong overall increase in H3K9me2 and H3K27me2 levels demonstrate the biochemical role of the demethylase activity in vivo. However, from this study, it is not possible to discriminate whether it is the increased H3K9me2 or H3K27me2 levels or both leading to the observed phenotype in *C. elegans*. Furthermore, although we do not have any evidence for a role of PHF8 in demethylating other methylated lysines than those present on H3K9 and H3K27, PHF8 and its *C. elegans* homolog might catalyze demethylation of other methylated lysines present on histones or other proteins in vivo.

It is striking to note that inactivation of PHF8 in humans and its *C. elegans* homolog gives relatively mild phenotypes, taking the large number of genes that are potentially regulated by PHF8 into account. This suggests that the PHF8 demethylases are not functioning as on-off switches for transcription but, rather, appear to fine-tune transcription, and their function is only important for the specific development of certain cell types. Alternatively, due to a potential redundant function of the other proteins of the PHF8 family and potential ZNF711 homologs, PHF8 and ZNF711 might contribute to many other cellular processes in addition to neural development. In support of this, we have shown that the PHF8 and ZNF711 bind to a large number of genes that are required for the proliferation and differentiation of many different cell types in addition to neural cells.

In conclusion, we have demonstrated that PHF8 catalyzes the demethylation of H3K9me2/me1 and that it associates with the active or poised H3K4me3-positive and H3K9me2-negative genes, including the XLMR gene *JARID1C*. Furthermore, our results suggest a role for the transcription factor and XLMR protein ZNF711 in the recruitment of PHF8 to a subset of target genes, which may stratify those involved in mental retardation from those involved in cleft/lip palate malformation. Finally, the *C. elegans* homolog *F29B9.2* of PHF8 is also an H3K9me2 demethylase, and consistent with this and the important role of PHF8 in neuronal function, inactivation of *F29B9.2* leads to uncoordinated locomotion.

## Experimental Procedures

### Cloning Procedures

The open reading frames (ORF) of human PHF8 and ZNF711 were generated by PCR amplification using a human fetal brain cDNA library (Invitrogen) as template. The ORF of *C. elegans F29B9.2* (cePHF8) was amplified from *C. elegans* cDNA. The amplified fragments were cloned into the pCR8/GW gateway entry vector (Invitrogen, Carlsbad, CA), and the DNA sequences were verified by sequencing. To generate expression vectors, the appropriate entry clones were transferred into gateway-compatible pCMV-HA, pCMV-Myc, baculovirus virus transfer vectors pVL-flag.his and pACGHlt-A, and pGEX for *E. coli* expression. shRNA constructs targeting PHF8 and ZNF711, sh*PHF8* (bp 1653–1671) were constructed in pFUGW-UbqC-HygroEGFP, and sh*ZNF711* in pLKO.1 was obtained from Sigma-Aldrich.

### Generation of Antibodies to PHF8 and ZNF711

Polyclonal antibodies were generated by immunizing rabbits with affinity-purified insect cell-produced GST-PHF8 (amino acids 1–548) or bacterially expressed GST-ZNF711 (amino acids 1–358). The antibodies were absorbed on GST-coupled cyanogen bromide-activated Sepharose (GE Healthcare) and subsequently affinity purified using Sepharose (GE Healthcare) coupled with GST-PHF8 or GST-ZNF711. Antibody specificity was tested and confirmed by immunoblotting and immunoprecipitation.

### Demethylation Assay

For the generation of recombinant proteins, please see the Supplemental Experimental Procedures. Calf thymus histones (Sigma Aldrich) or synthetic histone H3 peptides (ARTKQTARKSTGGKAPRKQLATKAARKSAPATGGVKKPHR-YC-Ttds-K-Biotin; JPT, Germany) mono-, di-, or trimethylated at the K9 or K27 position were incubated with purified Flag-His-PHF8, GST-PHF8 (amino acids 1–548) or Flag-His-cePHF8 for 60 min at 37°C in demethylation buffer (50 mM HEPES-KOH [pH 7.7], 250 mM NaCl, 1 mM MgCl_2_, 1 mM α-ketoglutarate, 40 μM FeSO_4_, and 2 mM ascorbic acid). Reaction mixtures were analyzed by either western blotting using specific antibodies (as listed in the Supplemental Experimental Procedures) or mass spectrometry. For mass spectrometry analysis, four micrograms of recombinant GST-PHF8 was incubated with 2.5 μg either H3K9me3, H3K9me2, H3K9me1, or H3K27me3 peptide in demethylation buffer in a final volume of 90 μl for 30 min at 37°C. Reaction mixtures were analyzed as previously described (Christensen et al., 2007).

### ChIP Assays

Chromatin immunoprecipitation assays (ChIP) were performed and analyzed as previously described (Bracken et al., 2006). A detailed protocol for ChIP-seq analysis and antibodies used for ChIP are included in the Supplemental Experimental Procedures.

## Accession Numbers

The genome-wide mapping data of H3K4me3, PHF8, and ZNF711 for SH-5YSY cells reported in the paper are available at the Gene Expression Omnibus server (http://www.ncbi.nlm.nih.gov/geo/) with the accession number GSE20673.

## Supplemental Information

Document S1. Supplemental Experimental Procedures and Five Figures
