# Supplementary material for: A Functional Link between the Histone Demethylase PHF8 and the Transcription Factor ZNF711 in X-Linked Mental Retardation
Source: Mol Cell. 2010 Apr 23;38(2-2):165–78. doi: 10.1016/j.molcel.2010.03.002 (PMC2989439; doi:10.1016/j.molcel.2010.03.002)
Supplement: Document S1. Supplemental Experimental Procedures and Five Figures [file mmc1.pdf]

Molecular Cell, ***Volume 38***

Supplemental Information

A Functional Link between the Histone Demethylase PHF8 and the Transcription Factor ZNF711 in X-linked Mental Retardation

Daniela Kleine-Kohlbrecher, Jesper Christensen, Julien Vandamme, Iratxe Abarrategui, Mads Bak, Niels Tommerup, Xiaobing Shi, Or Gozani, Juri Rappsilber, Anna Elisabetta Salcini, and Kristian Helin

Supplemental Experimental Procedures

Recombinant PHF8, ZNF711 and F29B9.2

Recombinant baculoviruses were generated by cotransfection of baculovirus transfer vector containing either a deletion mutant encoding N-terminally hexahistidine-tagged human PHF8 (amino acids 1-548) or full-length flag-his-tagged PHF8 or cePHF8 and Bsu36I linearized Bakpak6 baculovirus DNA· Histidine-tagged proteins were expressed in *Trichoplusia ni*, High Five, and purified by Cobalt-Sepharose (Clontech) or Gluthathione-Sepharose (GE Healthcare). Insect cells were incubated at 28°C and harvested 40–44 h post infection, washed twice in PBS, resuspended in 25 mM HEPES–KOH, pH 7.6, 5 mM KCl and 1.5 mM $MgCl_2$ and lysed by Dounce homogenization. After 20 min of incubation the lysates were adjusted to 300 mM NaCl and further incubated for 30 min and cleared by centrifugation for 30 min at 20 000 *g*. The supernatant was loaded on to a Cobalt–Sepharose column (Clontech) (1.5 ml of resin per 10^9 cells) equilibrated in buffer A (25 mM HEPES–KOH, pH 7.6, 0.5 mM $MgCl_2$, 0.5 mM DTT and 10% glycerol) adjusted to 300 mM NaCl. The column was washed with the same buffer and eluted with buffer A adjusted to100 mM imidazole. The eluted fractions were analysed by SDS–PAGE, flash frozen in liquid N_2, and stored at –80°C. All procedures were performed on ice or at 4°C in the presence of complete EDTA-free protease inhibitor (Boehringer Mannheim).

Peptide arrays and PHD finger mapping

Biotinylated histone peptides were synthesized at the Yale W.M. Keck facility as previously described (Shi et al., 2007). Peptides were printed in six replicates onto Streptavidin-coated slides (ArrayIt) using the VersArray Compact Microarrayer

(BioRad). All printed slides were air-dried overnight prior to use. Directly before use, unbound Streptavidin sites were blocked with free biotin (Sigma; 1mg/mL). Slides were incubated at 4°C overnight with GST-PHF8 PHD finger amino acids 1-65) diluted in peptide binding buffer (50mM Tris-HCl 7.5, 150mM NaCl, 0.1% NP-40, 20% fetal bovine serum). Slides were washed 6 times with peptide binding buffer and probed with anti-GST antibody (Millipore) diluted in PBS containing 0.1% Tween-20 (PBST) and 20% FBS at room temperature for 1 hour. Slides were washed with PBST six times, then incubated 30 minutes with Alexa Fluor 647 chicken anti-rabbit IgG (Invitrogen) diluted in (PBST with 20% FBS). Lastly, slides were washed with PBST 6 times, briefly rinsed with PBS and air-dried. A GenePix 4000 scanner (Molecular Devices) was used to scan the arrays, and data images were analyzed by GenePix Pro Version 56.0 1 software. Peptide pull-downs**:** biotinylated peptides (xx μg) were bound to streptavidin beads in binding buffer (50mM Tris-HCl, pH 7.5, 300mM NaCl, 0,2% Nonidet P-40, 1μM ZnSO4, 0.2mM PMSF, 1μg/ml aprotinin, 1μg/ml leupeptin) at 4°C. The beads were washed three times with binding buffer and then incubated with 1 μg of GST-PHF8 fusion protein or HeLa cell extract for 4 h at 4°C. After 4 washes with binding buffer, the beads were boiled in protein loading buffer, fractionated by SDS–polyacrylamide gel electrophoresis and subjected to western blot analysis using an anti-GST (Amersham 27-4577) or anti-PHF8 antibody.

PHF8 complex purification

In order to isolate PHF8 containing complexes, two-step affinity purification was performed followed by mass spectrometry analysis. Nuclear extracts (250-500mg, $3x10^9$ cells) from Flp-In^{TM}-T-REx^{TM}-293 cell lines expressing a Flag-HA tagged PHF8 was precleared with protein G Sepharose (GE Healthcare) for two hours and incubated with a 700 μl packed volume of anti-Flag-beads (Anti-Flag® M2-agarose, Sigma) overnight at 4 ˚C with rotation. The beads were collected by centrifugation at 700 x g for 5 min and washed 6 times with 40 x resin bed volume of buffer A (20mM Tris-HCl, pH 8.0, 300 mM NaCl, 1.5 mM $MgCl_2$, 0.2 mM EDTA, 10% glycerol, 0.2 mM PMSF, 1 mM DTT, 1 μg/ml aprotinin and 1 μg/ml leupeptin). The beads were transferred into a 10 ml poly-prep chromatography column (Bio-Rad) and complexes were then eluted five times after 10 min of incubation using one resin bed volume of buffer A supplemented with 0.5 μg/μl FLAG peptide. The eluate was subjected a

second round of purification using an antibody against the HA-tag. The Flag-IP elute was incubated with 200 µl of a 50 % slurry of HA-beads overnight. The beads were washed four times with buffer A and eluted with 100 µl buffer A supplemented with 1 µg/µl HA peptide for two hours. The samples were boiled in SDS loading buffer and run shortly into a SDS gel in order to remove the Flag and HA peptide and other contaminations. A gel slice containing the purified proteins was isolated for mass spectrometry analysis.

Protein interaction assays

For co-immunoprecipitation assays, Phoenix cells were transfected with expression vector pCMV-HA-PHF8 and pCMV-Myc-ZNF711. Cell lysates were prepared by sonication in E1A lysis buffer (250 mM NaCl, 50 mM Hepes, pH 7.0, 5 mM EDTA, 0.1% Nonidet P-40) and incubated with 5 ug of HA-, Myc or control antibody overnight at 4ºC. After incubation with protein G Sepharose for two hours, the immunoprecipitates were washed five times, boiled in SDS-sample buffer and analyzed by SDS-PAGE followed by western blotting. For immunoprecipitation of endogenous PHF8 and ZNF711, total lysates from HEK293 cells were used and incubated with 5µg of PHF8-, ZNF711- or control antibody.

To examine protein interactions in mammalian two-hybrid assays, human U2OS cells were seeded in 24-well tissue culture plates. Next day the cells were transfected with combinations of reporter plasmid, pGal-luc and pGal-ZNF711 and pVP16-PHF8 expression vectors and controls as described in the figure legends using Lipofectamine 2000 (Invitrogen) according to the manufacturer's recommendations. The vector pCMV-LacZ expressing β-galactosidase was included as an internal control and used for normalization of the luciferase activities. Cells were harvested 48 h after transfection, and β-galactosidase and luciferase activity were measured essentially as described previously.

Microscopy

Fluorescence microscope picture was acquired using an Axiovert 135, Carl Zeiss, Inc. with a 20 x Plan NEOFLUAR with a NA of 0.75. Pictures were taken at room temperature with a CoolSNAP cf2; Photometrics camera using MetaMorph software (MDS Analytical Technologies). Pictures were exported in preparation for printing using Photoshop (Adobe).

Real-time quantitative PCR (qPCR)

Total RNA was isolated from eggs using TRIzol® reagent (Invitrogen) and RNAeasy Minikit (Qiagen). cDNA was synthesized using reagents from the TaqMan Reverse Transcription kit (Applied Biosystems). qPCR was performed using SYBR Green 2x PCR Master mix (Applied Biosystems) in an ABI Prism 7300 Real Time PCR system (Applied Biosystems). The measures were normalized to ribosomal protein (rpl-26) RNA levels. All reactions were performed in triplicate, in at least three independent experiments.

Antibodies for detecting modifications in demethylation assays

Anti-H3K9me3 (Upstate 07-523), anti-H3K9me2 (abcam 1220), H3K9me (Abcam 8896), anti-H3K27me3 (Upstate 07-449), anti-H3K27me2 (Abcam 24684), anti-H4K20me3 (Upstate 07-463), anti-H4K20me2 (Upstate 07-747), anti-H3K4me3 (Abcam ab8580), anti-H3K4me2 (Upstate 07-030), anti-H3K36me3 (Abcam ab9050), anti-H3K36me2 (Upstate 07-369), anti-H3 (Abcam Ab1791-100).

ChIP assays, ChIP-seq and gene expression analysis

The antibodies used were specific for PHF8, ZNF711, H3K4me3 (Abcam 1012, Cell Signaling C42D8), H3K9me2 (Upstate 07-441), RNA PolII (Santa Cruz sc899), H3K9ac (Abcam 10812), H3K14ac (Upstate 07-353), H3K27me2 (Cell Signaling D18C8). For ChIP-seq of PHF8 and ZNF711 and for the different histone marks, 1mg and 0.5mg total protein was used, respectively. Subsequently the DNA was at Ethanol precipitated and adaptors were ligated. Finally, the DNA was amplified using a kit from Illumina (IP-102-1001) and analyzed by Solexa/Illumina high throughput sequencing. The sequence reads were aligned to the human genome (hg18) using the Illumina Analysis Pipeline allowing one mismatch. Peak detection, binding and gene annotation analysis were performed using the Cisgenome program (Ji et al., 2008) at an FDR cut-off value <0.1. IgG was used for normalization. Venn diagram analysis and Nucleotide overlap calculation was performed with Galaxy browser (www.galaxy.psu.edu).

For expression analysis, total RNA was purified from SH-SY5Y cells using RNAeasy (Qiagen). The RNA was reverse transcribed using a TaqMan reverse transcription reagents from ABI, according to the manufacturer's instructions. For RNA quantification, reversed-transcribed total RNA was analyzed by real-time PCR

using SYBR Green PCR Master Mix (Fermentas) and an ABI prism 7300 Sequence Detection system. All reactions were analyzed in triplicates. Primer sequences are included in supplementary information

Primers used in this study

qPCR

PHF8 fw 5-TCGAAAACTTGTGGCCAGAGG
PHF8 rv 5-AGAGGTGCCACCAAAGTCAATG

KIAA1718 fw 5-CCCATACCATTCCCGAAGAAAG
KIAA1718 rv 5-GCTCAAGATGTCCTCTTCCCATG

PHF2 fw 5-TGGAGCCACCTGACATTGTAAAG
PHF2 rv 5-CCTTCACGCAGATTAGGCAGTAC

ZNF711 fw 5-CACACGCCAGACTCTAGAATGG
ZNF711 rv 5-CCATTCCAGCCACAAAATCTTG

ZNF41 fw 5-TGCCCAGAGACGCCTGTAC
ZNF41 rv 5-CACTGAGAGCAGGTGGCTGTAG

JARID1C fw 5-TGCATAAGCTGAAGGTTCGGG
JARID1C rv 5-GCCACTCGCACTTTGTTGG

CDC40 fw 5-AGCAAAATCTCTTTGTGGCTGG
CDC40 rv 5-ACAATGGTGTTGACAGCTCCC

PCBP2 fw 5-TCATGACCATTCCGTACCAGC
PCBP2 rv 5-TCTAGAGGTGGTCCCTCCAGG

TAF6 fw 5-TACTACAAGGAGATCACCGAGGC
TAF6 rv 5-ATAAAGGTACTGAACCGTGGCAG

CDCA7 fw 5-TCTGTGACTGATTCCAACTCCG
CDCA7 rv 5-AGACATGAGTTTTGCAAGCATTG

C20orf34 fw 5-GAAGGCTGGTGTGTTTAAGACCC
C20orf34 rv 5-GGCAGTCAGCACACATAACAATG

CXXC1 fw 5-ACCTACCTCTGGATCCTGACCTG
CXXC1 rv 5-CATGCTTCACTTTCACTGCCC

USP31 fw 5-TTAAAATCTTGTGGACCAGGTGG
USP31 rv 5-GACGAACACTTTCTGCATCAGG

PTBP1 fw 5-TCCAAGTTCGGCACAGTGTTG

PTBP1 rv 5-CGTTGTAGATGTTCTGCCCGTC

MYST2 fw 5-AATGCGCCTTCTTCTGAGTCTG
MYST2 rv 5-GGCGATGAGAGAGATCACTGC

LRRC41 fw 5-AGGCCTTCCAGTTTGGAAAGTG
LRRC41 rv 5-CACAAAGACGCCTGTCAGAAGAC

ChIP analysis

JARID1C fw 5-TTTCTTCCAAACTGTGTGGTTGCC
JARID1C rv 5-TGGGGAGGGGAGACGCTGAC

ZNF41 fw 5-TTGGAAGGGCGGTCATTTCTG
ZNF41 rv 5- GGCGGAAGAGGCAAAGCG

ZNF81 fw 5-AGACCCCCTCCTCCAGTTAG
ZNF81 rv 5-GCTGGTGGCAGGGAGCACG

C2orf34 fw 5-GTAGCCCTGCCACAGCTCTCTC
C2orf34 rv 5- TTGCTGGTAGCCATCTTGAGTG

PCBP2 fw 5-GCTATGGCTACAATTTCCACTTTTG
PCBP2 rv 5-GCTCGTGACTAGGACCCGAG

PRMT1 fw 5-GATTCTGGACGCCAGAGTTGG
PRMT1 rv 5-GGAAGATTTCATTGGCAATGGAG

MyoB1 fw 5-CCACTGTTTGCCGGAATGTG
MyoB1 rv 5-CACGGAAGGCAGTCTTTCTACC

HOXB1 fw 5-CAAGACAGCGAAGGTGTCAGAG
HOXB1 rv 5-CGGCTCAGGTACTTGTTGAAATG

SYP fw 5-ATGCTGCTGCTGGCGGACGG
SYP rv 5-TGGCCACCACCTCCCAGAGTC

CC2orf34/1 fw 5-TTATCCATCCATCTTTCATCCCC
CC2orf34/1 rv 5-RCAGATCGCCTCATCCCTGG

C2orf34/2 fw 5-GTAGCCCTGCCACAGCTCTCTC
C2orf34/2 rv 5-TTGCTGGTAGCCATCTTGAGTG

CC2orf34/3 fw 5-CCTGCTCGCCTCACCTTTG
CC2orf34/3 rv 5-AGCTTCGCAGGTGGCTCC

CC2orf34/4 fw 5-GGTTCGAGTTACCGATATTCGTCTG
hCC2orf34/4 rv 5-CCGCTGTACGGAAGATGCC

JARID1/1 fw 5-GGATCCCTTTATGAATCTGATACAAGC
JARID1C/1 rv 5-CTGAGTCCCAAAGTCTTTAAGATGAGG

JARID1C/2 fw 5-TTTCTTCCAAACTGTGTGGTTGCC
JARID1C/2 rv 5-TGGGGAGGGGAGACGCTGAC

JARID1C/3 fw 5-ACCCCCCAAATCACTGAATTG
JARID1C/3 rv 5-TTATCCTCCTCAAGCTCCCAAAG

JARID1C/4 fw 5-AAGCACTGTCTTCCCCGTCTAAG
JARID1C/4 rv 5-AAAAAAAAGCAAAACAAGAAACTTGG

Western blot analysis

Protein extracts from *tm3713* and N2 strains were prepared from young adult animals grown on OP50 at 25°C. For RNAi-treated animals, extracts were prepared from F1 *eri-1* adults grown on HT115 containing either the empty feeding vector, or specific RNAi. Worms were washed 2 times in M9 buffer and resuspended in an appropriate volume of urea buffer (1% SDS, 9M urea, 25mM Tris-HCl pH6.8, 1mM EDTA, 0.7M ß-mercaptoethanol), boiled 10 min at 95˚C and sonicated 3 times for 10 seconds with a Digital Sonifier 450D (Branson) at 20% amplitude, and spun at 15000 rpm for 20 min. 5x Laemmli buffer (containing DTT) was added to supernatant before loading on 15% polyacrylamide gel (SDS-PAGE). Equal amounts of protein lysate (approximately 20µg) were loaded on the gel. The following antibodies were used: polyclonal anti-H3 (Abcam 1791) 1:30000; monoclonal anti-H3K9me2 (Abcam 1220) 1:1000, polyclonal anti-H3K27me2 (Abcam 24684) 1:2000 and peroxidase-labeled anti-rabbit and anti-mouse secondary antibodies (Vector) 1:10000.

***C.elegans* strains**

C.elegans strains were cultivated using standard conditions (Brenner, 1974). The Bristol strain (N2) was used as the WT strain. Other strains used: *eri-1(mg366)* IV, *F29B9.2(tm3713)* IV. The *C. elegans F29B9.2* gene is located on chromosome IV and encodes for two transcripts encompassing eleven and ten exons, coding for predicted proteins of 910 amino acids and 897 amino acids, respectively. The ATG of the gene is located at position 15142 bp of the F29B9 cosmid (U70849) and a TAG terminator codon at position 19074 bp. The *C.elegans* mutant strain *tm3713* has been isolated by the Japanese National BioResource Project. *tm3713* lacks 575 bp and the deletion is located in exons four and five at position 15767-16342 of the Genbank entry U70849.

The mutant gene is carrying an in-frame deletion encoding a putative protein of 773 amino acids and it is missing the region containing the PHD domain. The deletion was confirmed by sequencing the entire *F29B9.2* locus of the *tm3713* allele and RT-PCR analysis confirmed that the *tm3713* mutant gene with the predicted deletion is transcribed. The original strain *tm3713* obtained by the NBP-Japan showed a mec phenotype that however disappeared after extensive backcrossing. *tm3713* was backcrossed to N2 background animals at least six times before the phenotypic analysis here reported.

Construction of GFP-tagged F29B9.2

For the *F29B9.2::GFP* construct, a 4715-bp fragment, including 786 bp of promoter region and the entire F29B9.2 genetic locus, was PCR-amplified from N2 genomic DNA using the primers DK101 (ctgcagcagagtaattgagcaagcatg) and DK102 (tctagacacattttgagcttcttcat) containing a *Pst*I and a *Xba*I restriction site, respectively. The resulting fragment was inserted in the multiple cloning site of the pPD95.75 vector (Fire lab) after *Pst*I–*Xba*I digestion. The DNA sequence of the construct was verified by sequencing. The constructs carrying the *F29B9.2* gene fused to GFP and under specific promoters (*myo-3* and *rab-3*) were obtained using a Multisite Gateway three-fragment vector construction Kit (Invitrogen).

Microinjection and production of transgenic lines

To obtain lines carrying extra-chromosomal arrays, the *F29B9.2::GFP* construct (30ng/ul) was co-injected with odr-1::DsRed (30ng/ul) in wild-type N2 worms. *tm3713;F29B9.2::GFP* transgenic line *(tm3713R*) was generated by crossing. *tm3713R-prab-3* and *tm3713R-pmyo-3* were obtained by injection of the constructs with odr-1::DsRed (30ng/ul) directly in *tm3713* mutant animals.

RNA interference

RNA interference was obtained by feeding, carried out as described previously (Timmons et al., 2001). An EcoRI fragment, derived from the *F29B9.2* open reading frame (1-1051 bp), was cloned into the L4440 plasmid and the transformed HT115 bacteria were seeded in NGM agar plates. Eggs prepared by hypochlorite treatment or starved synchronized L1 from *eri-1* strain were added onto the plates and cultivated at 15ºC. Control animals were fed with bacteria carrying an empty L4440 vector. The

efficiency of mRNA down regulation was tested by qPCR. F1 progeny was analyzed by western blotting.

Locomotion assay

Individual staged adult animals (24h at 25°C after L4 stage) were transferred to fresh NGM plates seeded with OP50 bacteria and allowed to recover for 10-20 min before pictures were taken. Tracks were measured from the pictures using ImageJ software. The amplitude of the path and the distance between successive peaks in the path (wavelength) were measured and values were normalized versus N2. More then 15 animals of each genotype and more then 70 measurements (wavelength and amplitude) were analyzed. Of note the phenotype observed in *tm3713* animals is not due to increased length of the animal body compare to wild type (not shown).

Supplementary Figure

Figure S1

A

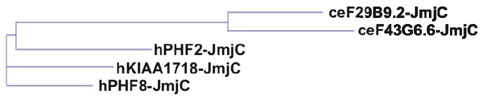

B

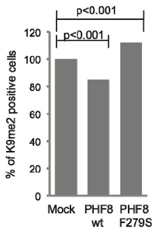

C

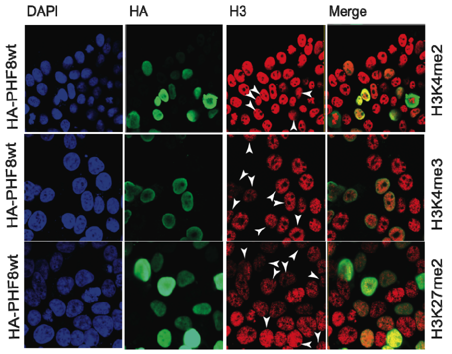

Supplementary Figures

Figure S2

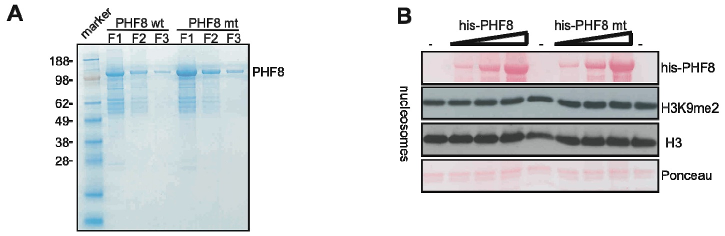

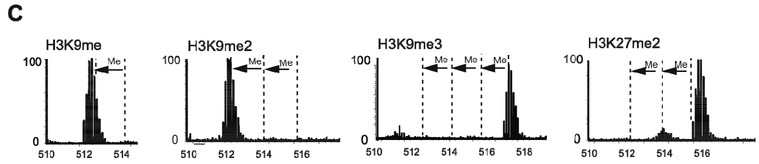

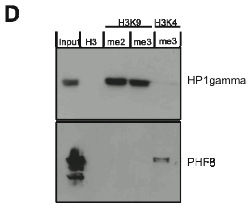

Supplementary Figures

Figure S3

A

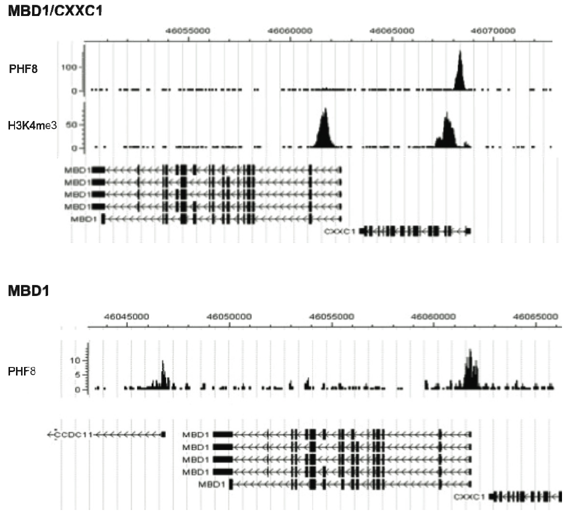

B

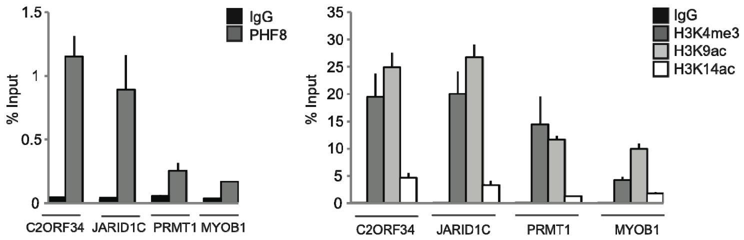

Supplementary Figure

Figure 4

A

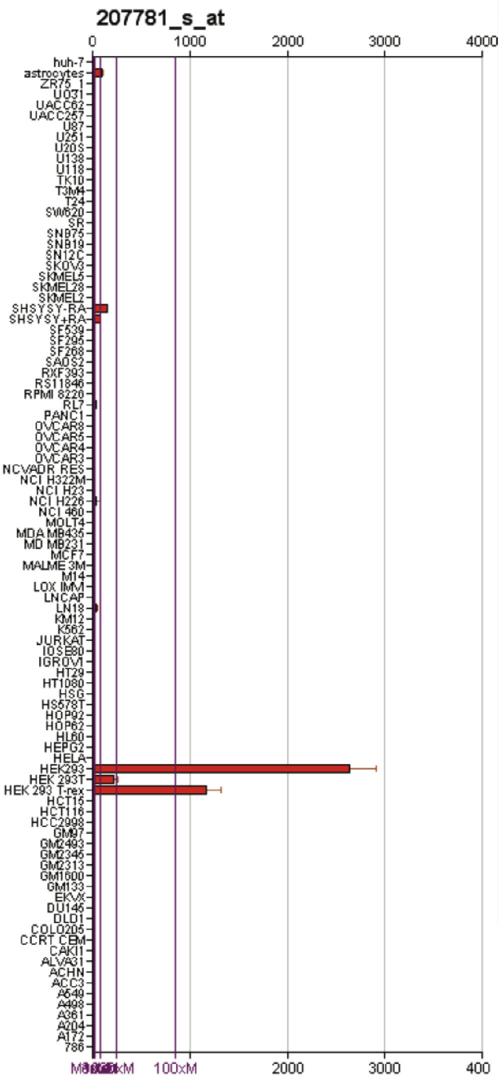

B

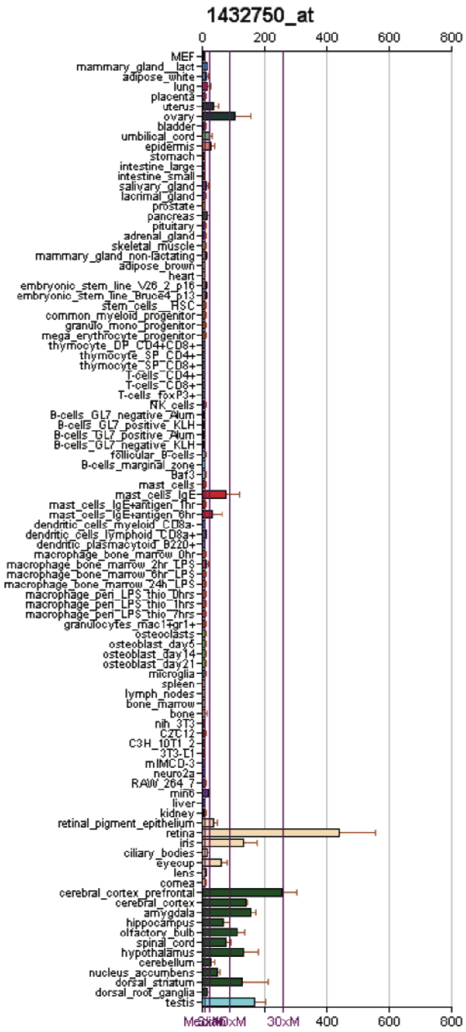

Supplementary Figure

Figure S4

C

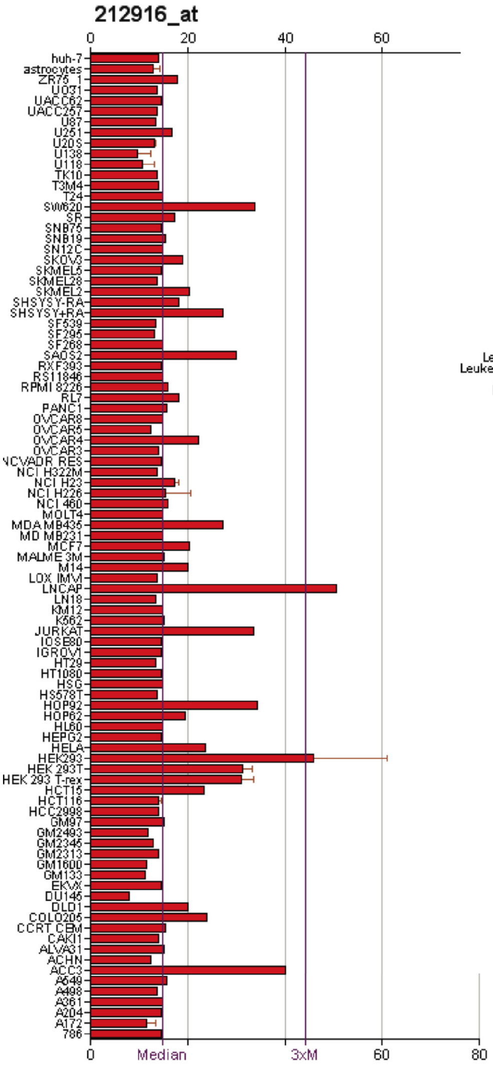

D

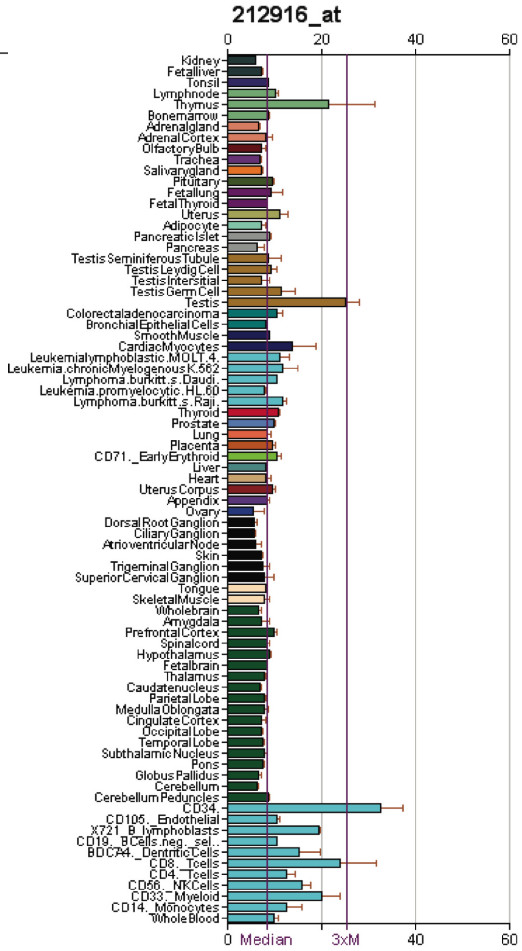

Supplementary Figure

Figure S4

E

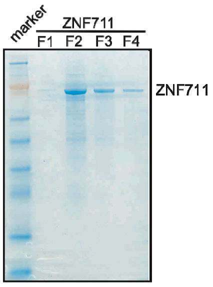

F

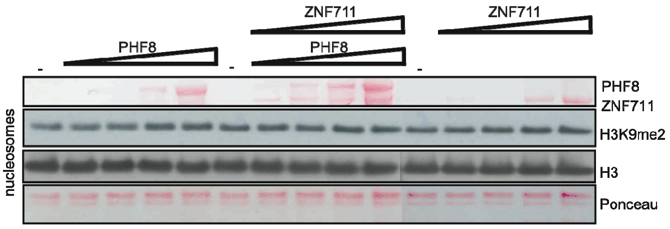

Supplementary Figure

Figure S5

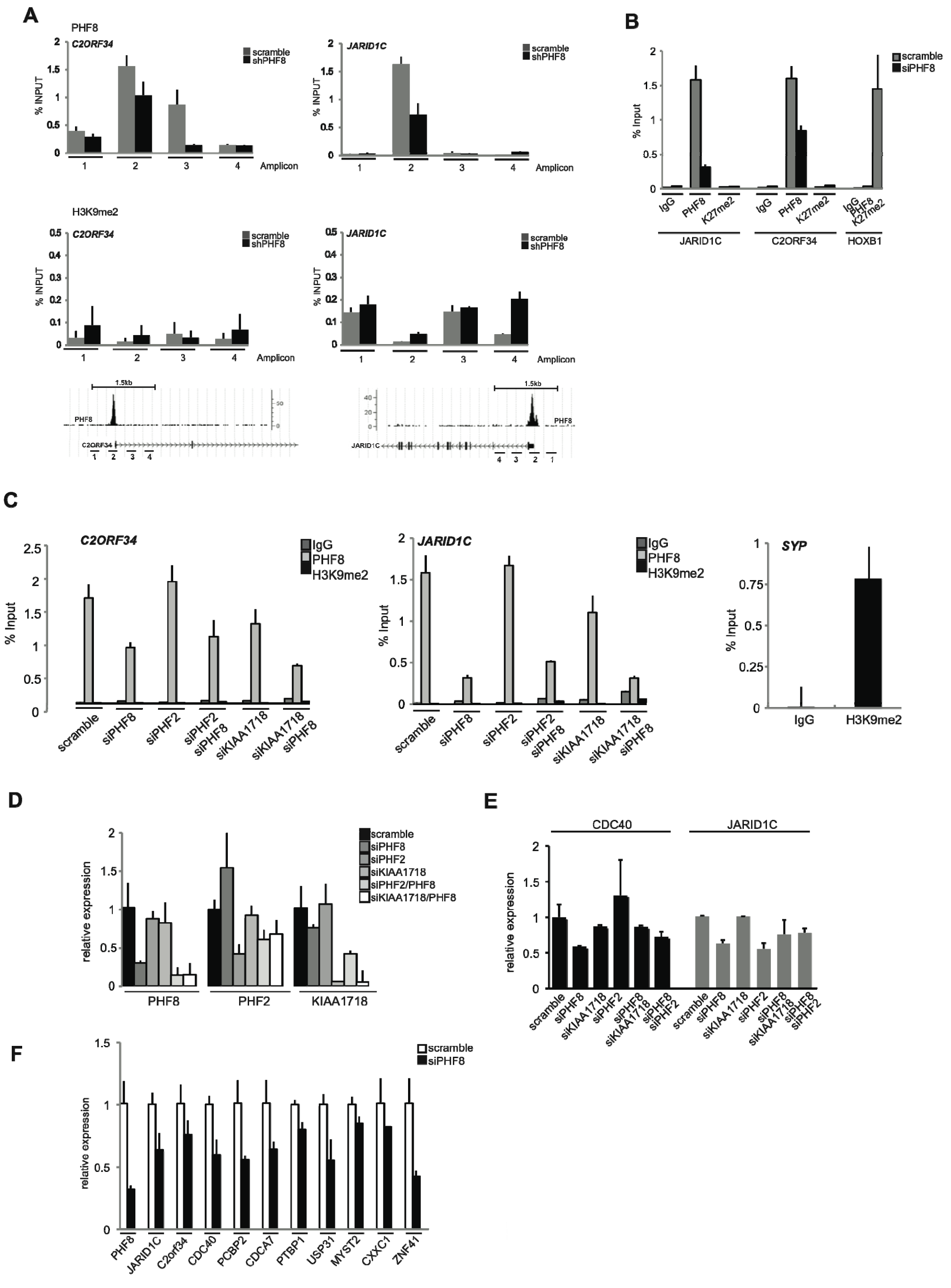

Supplemental Figure Legends

Figure S1. PHF8 demethylates H3K9me2 *in vivo*

(A) Phylogenetic analysis of the human and *C.elegans* PHF family. Alignment of the JmjC domains and construction of the phylogenetic tree were conducted using the neighboring-joining method (Saitou and Nei, 1987). **(B)** Quantification of H3K9me2 stainings for PHF8 wt and a PHF8 mutant. 293 cells were transfected with HA-tagged PHF8 wt and a XLMR mutant PHF8 (PHF8F279S). The transfected cells were fixed and co-stained for H3K9me2 and the expression protein (anti-HA). Images were acquired with an Incell 1000 high content imager (GE Healthcare) and analyzed using the Workstation 3.5 software. The Fishers exact test was used to calculate the significance. **(C)** 293 cells were transfected with HA-tagged PHF8. The transfected cells were fixed, and stained with antibodies recognizing the indicated histone modification and for the expression of the proteins (anti-HA), and analyzed by confocal microscopy. White arrows indicate cells expressing the tested protein. The cells were counterstained with DAPI to visualize cell nuclei.

Figure S2. PHF8 demethylates H3K9me2/me1 *in vitro* and binds to H3K4me3.

(A) SDS-PAGE analysis of purified His-tagged recombinant PHF8 and PHF8 JmjC mutant (H247R) expressed in insect cells. Marker is a molecular weight standard. Samples were subjected to SDS-PAGE and stained using Coomassie blue. The arrow indicates the position of recombinant PHF8 (120 kDa). **(B)** Demethylation assays of nucleosomes incubated with 4.5, 12.5 and 37.5 μg of recombinant PHF8; bottom panels show Ponceau stain of the nucleosomes/histones present in the assays. The middle panels show the reactions probed with the indicated antibodies and assayed by immunoblotting. **(C)** H3K9me3/me2/me1 and H3K27me2 peptides (2.5 μg) were incubated with saturating amounts of PHF8 (5μg) and analyzed by mass spectrometry. A shift in mass equivalent to one methyl group is indicated as "Me." **(D)** *In vitro* binding experiments. Hela cell lysate were incubated with biotinylated histone H3 peptides methylated at K4me3, K9me3 or K9me. After incubation with Streptavidin-agarose, the bound proteins were detected by western blotting using antibodies to the PHF8 and HP1γ (serving as a positive control for H3K9me3/me2 binding).

Figure S3. Correlation of H3K4me3 positive TSS and PHF8 binding to these sites (A) Binding profiles of the *MBD1* and *CXXC1* gene for PHF8 and H3K4me3 obtained from ChIP-seq analysis. The chromosomal locations are indicated according to hg18. The lower binding profile shows the chromosomal region of the *MBD1* gene for PHF8 enlarged. **(B)** qChIP analysis of *C2ORF34, JARID1C,PRMT1,* and *MYOB1* in SH-SY5Y cells using antibodies against PHF8, H3K9ac, H3K14ac and IgG control. The enrichment of the ChIP assays are shown as percentage of input. Error bars s.d., n=3.

Figure S4. Expression of ZNF711 in human cell lines and different mouse tissues. (A) mRNA expression analysis of ZNF711 in different human cell lines. The expression data was obtained from http://biogps.gnf.org. **(B)** Analysis of Znf711 mRNA expression in different mouse tissues. The expression data was obtained from http://biogps.gnf.org. **(C)** mRNA expression analysis of PHF8 in different human cell lines. The expression data was obtained from http://biogps.gnf.org. **(D)** Analysis of PHF8 mRNA expression in different mouse tissues. The expression data was obtained from http://biogps.gnf.org. **(E)** SDS-PAGE analysis of purified His-tagged recombinant ZNF711 expressed in insect cells. Marker is a molecular weight standard. Samples were subjected to SDS-PAGE and stained using Coomassie blue. The arrow indicates the position of recombinant ZNF711 (88 kDa). **(F)** Demethylation assays of nucleomes incubated with 1.5, 4.5 and 12.5 μg of recombinant PHF8 alone and/or in the presence of 1, 3 and 9 ug of recombinant ZNF711; bottom panels show Ponceau stain of the nucleosomes/histones present in the assays. The middle panels show the reactions probed with the indicated antibodies and assayed by immunoblotting.

Figure S5. No detectable changes in H3K9me2 and H3K27me2 levels on PHF8 target genes following PHF8, PHF2 and KIAA1718 downregulation.
(A) qCHIP analysis of *C2ORF34* and *JARID1C* in HEK293 cells after shRNA-mediated PHF8 knockdown using antibodies for PHF8, H3K9me2 and IgG control. Different primers pairs spanning a region 500bp up- and 1000bp downstream relative to transcription start sites were used to assess the binding of PHF8 (upper panels) and H3K9me2 (middle panels). The locations of the primers for both genes are illustrated in the lower panels. The enrichments obtained by ChIP are shown as percentage

bound of input. For panel A-F error bars s.d., n=3. **(B)** qChIP analysis of *JARID1C* and in SH-SY5Y cell after siRNA-mediated PHF8 knockdown using antibodies against PHF8, H3K27me2 and IgG control. Analysis of the gene coding for *HOXB1* was used as positive control for H3K27me2. The enrichments obtained by ChIP are shown as percentage bound of input. **(C)** qCHIP analysis of *C2ORF34* and *JARID1C* in SH-SY5Y cells cells after siRNA-mediated knockdown of PHF8, PHF2 and KIAA1718 using antibodies for PHF8, H3K9me2 and IgG control. The enrichments obtained by ChIP are shown as percentage bound of input. Analysis of the gene coding for Synaptophysin (SYP) was used as positive control for H3K9me2. **(D)** qPCR based expression analysis of PHF8, PHF2 and KIAA1718 in SH-SY5Y cells after siRNA-mediated knockdown. **(E)** qPCR expression analysis of PHF8 target genes *CDC40* and *JARID1C* after siRNA-mediated PHF8, PHF2 and KIAA1718 knockdown in SH-SY5Y cells. **(F)** qPCR expression analysis of PHF8 target genes after siRNA-mediated PHF8 knockdown in SH-SY5Y cells.

Supplemental References

Brenner, S. (1974). The genetics of Caenorhabditis elegans. Genetics *77*, 71-94.

Ji, H., Jiang, H., Ma, W., Johnson, D.S., Myers, R.M., and Wong, W.H. (2008). An integrated software system for analyzing ChIP-chip and ChIP-seq data. Nat Biotechnol *26*, 1293-1300.

Saitou, N., and Nei, M. (1987). The neighbor-joining method: a new method for reconstructing phylogenetic trees. Mol Biol Evol *4*, 406-425.

Shi, X., Kachirskaia, I., Walter, K.L., Kuo, J.H., Lake, A., Davrazou, F., Chan, S.M., Martin, D.G., Fingerman, I.M., Briggs, S.D*., et al.* (2007). Proteome-wide analysis in Saccharomyces cerevisiae identifies several PHD fingers as novel direct and selective binding modules of histone H3 methylated at either lysine 4 or lysine 36. J Biol Chem *282*, 2450-2455.

Timmons, L., Court, D.L., and Fire, A. (2001). Ingestion of bacterially expressed dsRNAs can produce specific and potent genetic interference in Caenorhabditis elegans. Gene *263*, 103-112.
